# The Effect of the Improvement Technology on the Quality of Midu Pork Roll

**DOI:** 10.3390/foods11223684

**Published:** 2022-11-17

**Authors:** Xue Xiao, Bowen Wang, Ping Zhao, Changrong Ge, Shijun Li, Zhichao Xiao

**Affiliations:** 1 Yunnan Engineering Technology Research Center for Processing of Livestock Products, Yunnan Agricultural University, Kunming 650201, China; 2 College of Foreign Languages, Yunnan Agricultural University, Kunming 650201, China; 3 School of Foreign Languages, Yunnan Agricultural University, Kunming 650201, China; 4 Yunnan Agricultural University, Kunming 650201, China

**Keywords:** Midu pork roll (MPR), quality analysis, physical and chemical indicators, free amino acids, volatile compounds

## Abstract

Midu pork roll (MPR), produced in Midu County, Dali Bai Autonomous Prefecture, Yunnan, China, is a traditional fermented meat product with a long history. This study aims to enhance the physical and flavor profile of MPR by improving its process, fermentation conditions and formulations. There were three different formulations, including traditional craft (control group: C), optimization process of Sichuan spicy flavor formula (Test group 1: T1) and optimization process of halogen flavor formula (Test group 2: T2). Higher moisture content, L*, a* and b* values and lower hardness, chewiness and shear force were observed in T1 and T2 compared to C (*p* < 0.05). A total of 15 free amino acids were detected throughout the fermentation process, during which the content of umami amino acids, sweet amino acids and bitter amino acids underwent significant changes. A total of 88, 85 and 75 volatile compounds were detected in C, T1 and T2, respectively, in which the relative content of alkanes and ketones in T1 and T2 were higher than those in C (*p* < 0.05). The process and formulas have improved the color, texture characteristics and tenderness of MPR to a certain extent, meanwhile, they have enhanced the flavor of MPR.

## 1. Introduction

Midu pork roll (MPR), traditional fermented meat produced in Midu County, Dali Bai Autonomous Prefecture, Yunnan province, China, has a longer shelf life than fresh meat products. This product has been included in royal recipes for more than 300 years since the Qing Dynasty (1636–1912). The traditional fermentation process of MPR is: (1) put an appropriate amount of fresh pork into a bag made from the skin of the feet of pigs; (2) add some ingredients into the bag, i.e., salt, red yeast rice and a variety of natural spices; (3) tightly seal the bag and cook it; (4) the pork meat in the bag will undergo fermentation under anaerobic conditions for 20 d. It is named roll hoof because its shape looks like the fresh feet of a pig [1]. The processed MPR is favored by local people for its nutritional value and non-greasy characteristics. For mass consumers, however, the strong sour taste, hard texture and single taste of MPR have become increasingly unacceptable. This, in fact, has restricted the development of MPR products.

Flavor is an important quality attribute for determining consumer acceptance and preference [2]. Fermentation conditions, the types of raw and auxiliary materials and the ratio of ingredients have an important influence on the formation of the characteristic flavor of MPR. Therefore, the degradation of meat protein, the oxidation of lipids and the Maillard reaction generated by heating, the rich short peptides and free amino acids (FAAs) are all important factors that determine the flavor of MPR. The amino acid metabolites produced during protein degradation account for 6.00% of the total volatile compounds [3]; the hydrolysis and oxidation of lipid substances generate about 60% of the volatile compounds in fermented meat products [4,5].

Headspace solid-phase microextraction gas chromatography-mass spectrometry (HS-SPME-GC-MS) has been widely used recently to identify volatile compounds [6,7,8]. FAAs, color, texture and water activity (Aw) are widely used in meat quality analysis. These factors are important indicators for distinguishing flavor profiles and characterizing differences in meat quality [9,10]. Hence, HS-SPME-GC-MS combined with FAAs analysis and physical and chemical indicators analysis can be used to characterize the flavor and the quality of MPR under different process conditions, which may also provide a reference for establishing a much more comprehensive, quick method to identify those key factors of MPR.

Based on the traditional production method of MPR, this study specifically improved the process flow by controlling the fermentation conditions, changing the steaming time, adjusting the fat-to-lean ratio of meat, adding flavor enzymes and enzymatic tenderization technology and tumbling technology. In this way, the aim of this study is to improve MPR’s sensory quality and flavor, increase consumer acceptance, and also increase the economic value of MPR production. It provides a theoretical basis and more references for the development of traditional fermented meat products from different regions.

## 2. Materials and Methods

### 2.1. Materials

Pork loin, fat pork and spices (e.g., table salt) were purchased from Kunming Himat Supermarket (Kunming, China). The earthen pottery altar (diameter: 28 cm) was purchased from Shu rong Pottery Factory (Chengdu, China).

### 2.2. Preparation of MPR

Traditional craft (Control group: C): The feet of pigs were boned and preserved to make a feet skin bag, then the lean pork meat was cut into long irregular strips to fill the bag after mixing with spices. Meanwhile, the mouth of the bag was stitched and cooked in boiling water for 3 h and pickled and fermented with shredded radish at room temperature for 20 d. When the bag was cool, the MPR product was ready.

Optimization process: First, the pork meat was prepared, the pork loin was cut into 1 cm^2^ × 10 cm strips, then the pork fat was chopped into fat mash and 0.035% papain was added to tenderize the cut lean meat strips for 10 min. Next, the brine containing 0.15% spice was mixed with the pork meat. The mass ratio of tenderloin to fat was 8:2; this was kept at 4 °C in a refrigerator. After 24 h, the cured meat was kneaded for 0.5 h, then filled into the large piece of pig skin, which was steamed for 40 min, and finally tied with foil cotton rope and steamed for 20 min to set the shape and inactivate the enzyme. Finally, the MPR product was marinated and fermented with shredded radish at 14 °C for 10 d.

The experiment group was divided into two groups. Test group 1 (T1) used the improved technology of Sichuan spicy flavor formula and test group 2 (T2) used the improved technology of halogen flavor formula. The specific formulas are shown in Table 1.

### 2.3. Determination of Physical and Chemical Indicators

#### 2.3.1. The Cooking Loss (CL)

The CL was determined according to the American Meat Science Association [11]. The CL suggests the loss of MPR occurring in the cooking process. Sample 1 (W1, g) of MPR was measured before cooking, then sample 2 (W2, g) was measured after all the cooking steps were completed. The cooking loss rate is calculated using the following formula:

$$\mathrm{Cooking}\;\mathrm{loss}\;\mathrm{rate}\;\left(100\%\right)=\left(W1-\;W2\right)/W1\times 100\%$$

#### 2.3.2. The Yield of MPR

The yield of MPR refers to the ratio of MPR quality both before steaming and after fermentation. Sample 1 (S1, g) was measured before MPR was bundled and steamed. After the fermentation was complete, the sample record (S2, g) was determined, and the finished product was calculated using the following formula rate:

$$\mathrm{Yield}\;\left(100\%\right)=\;S2/S1\times 100\%$$

#### 2.3.3. pH (Acidity) Determination

The pH of MPR was measured by a pH meter according to AOAC [12]. The homogenized sample was leached with nine times the volume of distilled water for 30 min and then filtered, and the filtrate was measured with an acidity meter.

#### 2.3.4. Water Activity (Aw) Measurement

An Aw analyzer was used to determine the Aw in MPR samples [13]. 

#### 2.3.5. Moisture Determination

The moisture content was measured according to AOAC procedures [13].

#### 2.3.6. Chromaticity Assay

Coordinates L*, a* and b* values were measured at three different points on the sample, which were taken from the cross-section of the product, and triplicate measurements were averaged for each coordinate per test group. These measurements were performed by using a colorimeter that was previously calibrated with the CIELAB system. L* represents lightness (L* = 0 black, 100 white); a* (redness) ranges from green (−) to red (+); and b* (yellowness) ranges from blue (−) to yellow (+). Measurements were made at a 2° viewing angle by using illuminant C [14]. Three readings were performed on each sample to obtain a mean value for each test group.

### 2.4. Texture Determination

The determination of texture was performed using the Texture profile analysis (TPA) texture analysis method [15]. A section of the MPR sample was cut into three parts with the same specification (20 cm^3^ cube sample), five different points of each section were selected for measurement and each point was repeated three times. Finally, the average value was used as the result.

Tenderness is expressed by the value of shearing force (Warner–Bratzler shear, 1 cm^3^ cube sample) in parallel. For each sample, three replicates were performed, and the average value was used for statistical analysis.

### 2.5. Determination of FAAs

The measurement of FAA content referred to the method from Luo et al. [16]. FAA contents were detected by an automatic amino acid analyzer (S-433D, SYKAM Ltd., Munich, Germany).

A total of 5 g of the chopped meat sample was hydrolyzed in 15 mL of 6 M HCl solution. Then, 1.0 mL of the filtered hydrolysate was isolated, and 1 mL of 8% sulfosalicylic acid solution was added. The mixture was shaken well and rested for 10 min, then centrifuged at 10,000 rpm for 10 min at 4 °C. The supernatant passed through a 0.22 μm filter membrane, followed by quantification of the FAAs levels using an automatic amino acids analyzer.

### 2.6. Volatile Compounds Analysis 

The volatile compounds of MPR were extracted using SPME and analyzed using a GC-MS system (GCMS-QP2020 NX, Shimadzu Corporation, Kyoto, Japan) according to the method of Wen et al. [17].

After equilibration of 3 g of the sample in a 10 mL extraction flask at 60 °C for 5 min, 65 µm of PDMS/DVB (Polydimethylsiloxane/Divinylbenzene) fiber was inserted into the flask (1 cm from the sample) for the adsorption of volatile compounds at 60 °C for 40 min. Finally, the SPME device was inserted into the GC injection port for 5 min in splitless mode at 250 °C to allow complete desorption.

The GC conditions were as follows: HP-5MS capillary column (30 m × 0.25 mm × 0.25 mm; Agilent); carrier gas: He; flow rate: 1.0 mL/min; column oven temperature: 35 °C for 5 min, 2 °C/min to 60 °C, maintained for 3 min, and finally increased to 130 °C at a rate of 4 °C/min, held for 3 min and post-run at 280 °C for 3 min. The MS conditions were as follows: voltage 70 eV, ion source temperature 200 °C, mass scanning range *m*/*z* 33–600 and electron ionization (EI) mode.

The mass spectrum of the compound was compared with the NIST17. L mass spectrum database and the compounds with a matching degree greater than 80% were extracted and analyzed. The peak area normalization method was used to calculate the relative content of each component.

### 2.7. Sensory Evaluation

Sensory evaluation (i.e., color, texture, flavor and overall acceptability) was carried out concerning all samples experimented with by different processing techniques. Flavors were evaluated by 10 trained panelists. The panelists received training in the attributes, and the scale was used according to the method proposed by the ISO 6658:2017 regulation [18]. After training, the basic operation of sensory evaluation and the quality standards of different attributes must be mastered by panelists. A “warm-up” sample at the beginning of each session was evaluated to acquaint panelists with the scoring system. MPR slices (2 mm thick) were served randomly on white plastic plates coded with randomized three-digit numbers. The assessment of each attribute was determined as the mean value of all the tasters using an arbitrary scale of 1 (low) to 9 (high). The panelists were informed of the risks and nature of this study and were asked to give their consent to participate in the sensory analyses.

### 2.8. Data Analysis

#### 2.8.1. Statistics

All data were analyzed by SPSS software (SPSS 19.0, IBM, Inc., Armonk, NY, USA) and expressed as “mean ± standard” deviation. One-Way ANOVA analysis of variance and Duncan’s multiple comparison were performed for each group; *p <* 0.05 was the standard for the significant difference test; the drawing of experimental graphs was performed using Origin 9.0 (OriginLab Corp., Northampton, MA, USA).

#### 2.8.2. Taste Activity Value Analysis (TAV)

The TAV is the ratio of the measured value of the taste substance in the sample to its taste threshold. The calculation formula is as follows [19]:

TAV = ρ1/ρ2

In the formula: ρ1 was the mass concentration of the taste amino acid, mg/g; ρ2 was the taste threshold mass concentration of the amino acid, mg/g.

TAV can reflect the contribution of a single compound to the overall taste. In the case of TAV > 1, the substance is considered to contribute to the taste. When TAV is less than 1, it is considered that the substance contributes little to the taste, and thus the main taste amino acids of MPR are determined [20].

#### 2.8.3. Principal Component Analysis (PCA) and Comprehensive Evaluation

SIMCA 14.1 software (Sartorius Stedim DataAnalytics AB, Umeå, Sweden) was used for PCA to compare the differences between FAAs and volatile compounds under different process conditions. The major flavor compounds were screened and analyzed by the PCA and PLS-DA (Partial Least Squares Discriminant Analysis). The variable importance in projection (VIP) score was used to measure the degree of influence of each metabolite accumulation difference by the classification of each sample and its explanatory power. VIP ≥ 1 is the screening standard for common differential metabolites. Principal component information was used to comprehensively evaluate MPR under different process conditions [21]. A heat map was made using Origin to assess differences in volatile compounds.

## 3. Results and Discussion

### 3.1. The Influence of Process Optimization on the Physicochemical Analysis

The acidity of MPR has a great impact on its overall flavor. A lower pH value will reduce the sensory score of the product [22,23]. As is shown in Table 2, the pH of the T1 and T2 were 5.84 and 5.79, respectively, and for C, processed by traditional technology formula, it was 4.26, which was due to the long fermentation during the traditional process. The sour taste mainly came from lactic acid bacteria, which was influenced by the fermentation temperature and time [24]. For the traditional MPR, it is the long fermentation time that causes low pH, and many consumers can hardly accept it. Therefore, in this study, the fermentation time and temperature of test groups were adjusted to change the pH of MPR.

From a practical point of view, Aw measures the amount of water available for microbial metabolism, which controls the pathogen and spoilage microorganism growth and reproduction [25]. Wang et al. [26] reported that products have a longer shelf life and a higher quality with lower Aw. As Table 2 shows that the improved process and formulas had no effect on the ratio of free water and combined water of MPR. The study reported that the appropriate range of Aw along the fermentation and ripening processes is considered fundamental to achieving product stability [27]. The Aw values of the final MPR were slightly higher than those described by other authors [28,29]. However, after sterilization and modified atmosphere packaging, it is expected that a slightly higher Aw will not affect the shelf life of MPR.

The differences in water content, cooking loss rate and yield were used to compare the quality differences of MPR in three groups. Water content, cooking loss rate and the yield values of the three samples were significantly different (*p* < 0.05). The differences were mainly attributed to the addition of sodium hexametaphosphate and sodium alginate, water retaining agents, which played an important role in the improved process. The addition of the water-retaining agent improves the water holding capacity of MPR, reduces the loss of product nutrients and increases product quality. The results are consistent with those from Choi et al. [30], who reported that adding phosphate to cured meat products could increase water binding, thereby improving the product yield and reducing the cooking loss.

Color, as a key parameter for understanding images and describing objects, was essential in areas such as food quality assessment and real-time monitoring of food processing [31]. The color of meat is associated with several factors, such as pH and the iron state of pigment [32,33]. In this study, the L* value of T1 and T2 were significantly lower than C (*p* < 0.05), whereas the a* and b* values of T1 and T2 were higher than C (*p* < 0.05). Raw meat was kneaded for 0.5 h to make the red yeast rice pigment and meat fully mixed, which would have a certain effect on the bright red color of MPR.

### 3.2. The Influence of Process Optimization on the Texture of MPR

The texture properties of MPR in different groups are shown in Table 3. Significant differences were found in texture between C, T1 and T2. Hardness, cohesion, chewiness and shearing force in C were all significantly higher than in T1 and T2 (*p* < 0.05). Studies have found that the addition of exogenous enzymes during the processing of meat products can accelerate the degradation of flavor precursors in a short time and cause the structure to break [34]. When protein is degraded, it can lead to physical, chemical and structural changes in the myofibril. Muscle fibers and tendons were broken down and the cellular structure integrity was degraded. Subsequently, this improved the tenderness of the meat [35]. Usually, a reduction in hardness is considered positive in fermented meat [36]. Meanwhile, the addition of fat could significantly improve the rheological properties and cohesion of MPR so that MPR from the new process had a smooth taste [37].

### 3.3. The Effect of Process Improvement on the Content of FAAs of MPR

FAAs play a very important role in the flavor of MPR. They are important flavor substances, and certain amino acids are important flavor precursors [38]. In the process of MPR fermentation, FAAs are mainly derived from the hydrolysis of endogenous tissue enzymes (cathepsin and calpain) and the action of microorganisms and enzymes, which enhances the flavor and nutrition of fermented meat [39,40].

In this study, FAAs enhanced the flavor of MPR with a certain effect. Table 4 lists the content of each FAA in the C, T1 and T2 samples. The content of total free amino acids (TFAAs) in T1 and T2 is significantly higher than that of C (*p* < 0.05). The content of Glutamic acid (Glu) and Aspartic acid (Asp) in T1 and T2 are significantly higher than C (*p* < 0.05). The increase in TFAAs and umami amino acids in T1 and T2 may be related to the papain enzymatic hydrolysis process, and the addition of 1.50% protein isolate [34]. Meanwhile, the addition of excipients also had a certain impact on the composition and content of FAAs in MPR [41].

FAAs generated from protein decomposition can undergo the Maillard reaction and the Strecker amino acid degradation reaction to form volatile compounds, such as aldehydes, ketones, esters and pyrazines [42]. The content of FAAs in a sample depends on microorganisms and endogenous enzymes to hydrolyze proteins. The addition of enzymes and process improvements had a greater impact on the composition of FAAs.

### 3.4. Evaluation of the TAV of FAAs 

FAAs are one of the main base flavor substances in food. According to the taste characteristics of FAAs, they can be divided into four categories: umami amino acids, sweet amino acids, bitter amino acids and aromatic amino acids [43].

This study evaluated the TAV of FAAs before and after the improved process. TAV was introduced to determine the taste contributions of FAAs because FAAs with taste activity contribute to the taste of fermented food. A substance with TAV > 1 was considered an active component affecting the taste perception of the sample [44]. Consistent with a previous study, among all the FAAs, Glu contributed to the overall taste of MPR [45]. These FAAs are an important part of the formation of flavor substances in fermented MPR, which means they can be used as flavoring agents and nutritional supplements. 

The results are shown in Table 5. By the comparison of taste amino acids, the content of umami amino acids and bitter amino acids is higher than the others. As shown in Table 4, the umami amino acids of T1 and T2 were significantly higher than those of C. Among them, the TAV value of Glu was the highest (C: 2.56, T1: 4.97, T2: 5.10). Research by Yamaguchi et al. [46] found that Glu and Asp were the basic components of umami taste. Glu had the strongest umami taste, with a taste threshold of 0.3 mg/mL.

There was no significant difference in the TAV value of sweet amino acids between the three groups. Sweet amino acids can mask bitterness and astringency. Furthermore, they have a synergistic effect with umami amino acids to enhance flavor and freshness [47]. The TAV of Met in bitter amino acids was the highest. Its taste-presenting effect was that T1 and T2 were lower than C.

### 3.5. Principal Component Analysis of Free Amino Acids

**Figure 1:**
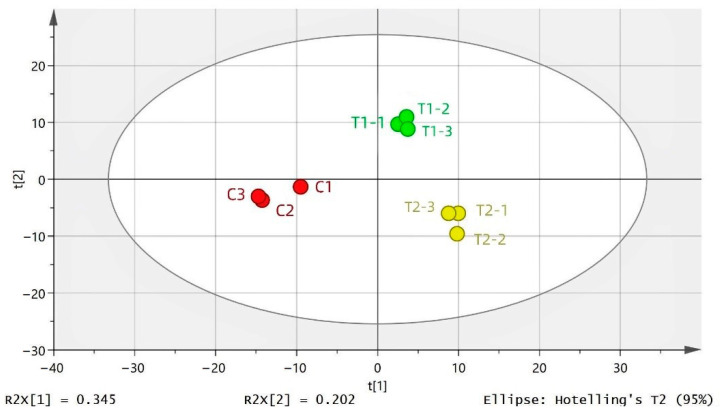
PCA results of FAAs in MPR samples with different processing. C: Control group; T1: test group 1 (using the improved technology of Sichuan spicy flavor formula); T2: test group 2 (using the improved technology of halogen flavor formula).

PCA was established to explore the differences in the FAAs in the samples from different processes and to gain an in-depth understanding of the changes in these substances during MPR fermentation. In Figure 1, the distinguishing effect of different samples is more evident, and the data points of MPR are all within the 95% confidence interval.

### 3.6. The Effect of Process Improvement in Various Volatile Compounds of MPR

**Figure 2:**
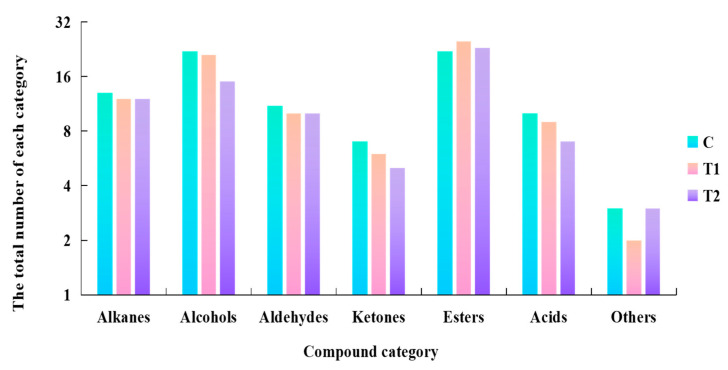
The total number of volatile compounds in three groups. It showed the total number of alkanes, alcohols, aldehydes, ketones, esters, acids and others in the three groups. C: Control group; T1: test group 1 (using the improved technology of Sichuan spicy flavor formula); T2: test group 2 (using the improved technology of halogen flavor formula).

The composition of volatile compounds of MPR is shown in Figure 2. A total of 88 volatile compounds, including alkanes (13), alcohols (22), aldehydes (11), ketones (7), esters (22), acids (10) and other volatile compounds (others, 3), were identified in C. A total of 85 volatile compounds were detected in T1, including (12) alkanes, (21) alcohols, (10) aldehydes, (6) ketones, (25) esters, (9) acids and others (2). A total of 75 volatile compounds were detected in T2, including (12) alkanes, (15) alcohols, (10) aldehydes, (5) ketones, (23) esters, (7) acids and (3) others. The results are consistent with those of Zhong et al. [24] and Chen et al. [48]. They reported that as the fermentation process progressed, a large number of flavor precursors were generated via carbohydrate metabolism, lipid oxidation, amino acid catabolism and bacterial esterification, resulting in a rapid increase in the types of volatile compounds in the MPR. Therefore, the composition of volatile flavor substances in C was more abundant.

Carbohydrate metabolism has a large effect on the flavor formation of MPR, which is primarily performed to generate acids, alcohols and ketones [49]. As shown in Table 6, the highest relative content of the three groups was alcohol compounds. First, wine was added during processing. At the same time, MPR products contain fatty acids, and alcohol is produced during the degradation of fatty acids, which is a very important flavor substance in meat products. In particular, more fat was added in the T1 and T2 groups, which further promoted the formation of fatty acids and their conversion to alcohol.

Most acids and aldehydes, such as octanoic acid, octanal, hexanal and nonanal, commonly originate from lipid autoxidation and play significant roles in the flavor development of fermented meat [50]. Due to their low threshold, aldehydes greatly contribute to the overall flavor; however, hexanal can lead to rotten odors, while nonanal and other linear aldehydes contribute to the flavor of fermented meat [24,51]. 

The relative contents of alkanes and ketones in C were lower than those in the test groups (*p* < 0.05). According to existing studies, alkane flavor compounds have a higher aroma threshold in meat products, and they have a significant flavor contribution only when the compound concentration is higher [52]. Amino acid catabolism esterification and Strecker reaction were also the major pathways for producing volatile compounds [53]. Among them, the α-keto acids, known as the important intermediates from the reaction of amino acid with dicarbonyl compounds, are metabolized to form the corresponding aldehydes; the aldehydes can also be converted into the corresponding alcohols and acids, which may provide fruity, malty and sweaty odors in the MPR [40].

Maybe due to the esterification [54,55], the lower contents of ester were found in T1 and T2 treatments compared to C (*p* < 0.05). Bacterial esterification of fatty acids with alcohols greatly affects the formation of esters that provide the characteristics of fruity and sweet odors and mask the rancid odor of meat products [56,57]. Ethyl esters were the most abundant esters in MPR in the present study, which may be derived from the esterification of ethanol and acids [58]. It was found that with the extension of fermentation time, the content of esters increased, but at the same time, acids would also increase [24]. Therefore, the flavor of fermented meat is the result of the comprehensive action of various substances.

### 3.7. Multivariate Statistical Analysis of Volatile Components

**Figure 3:**
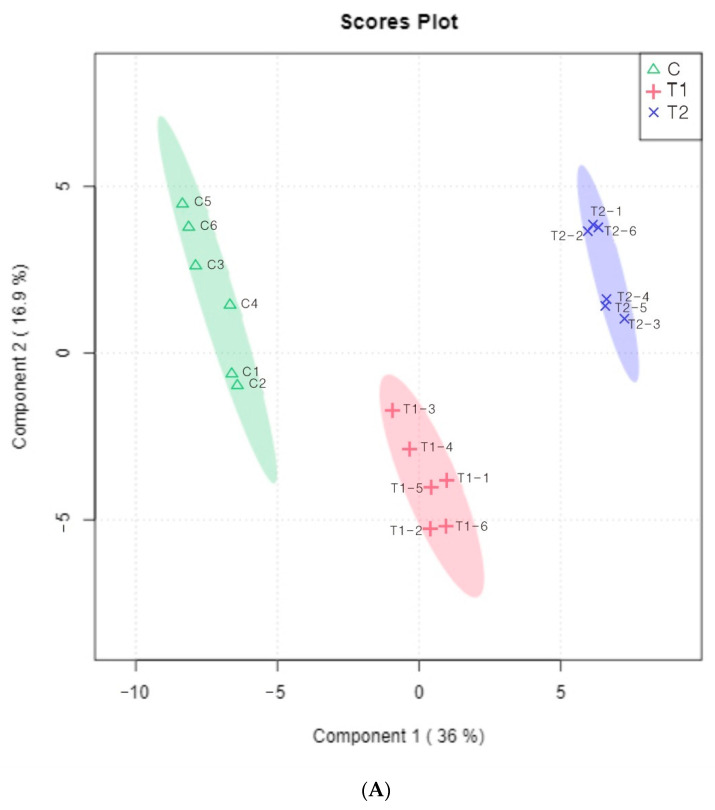

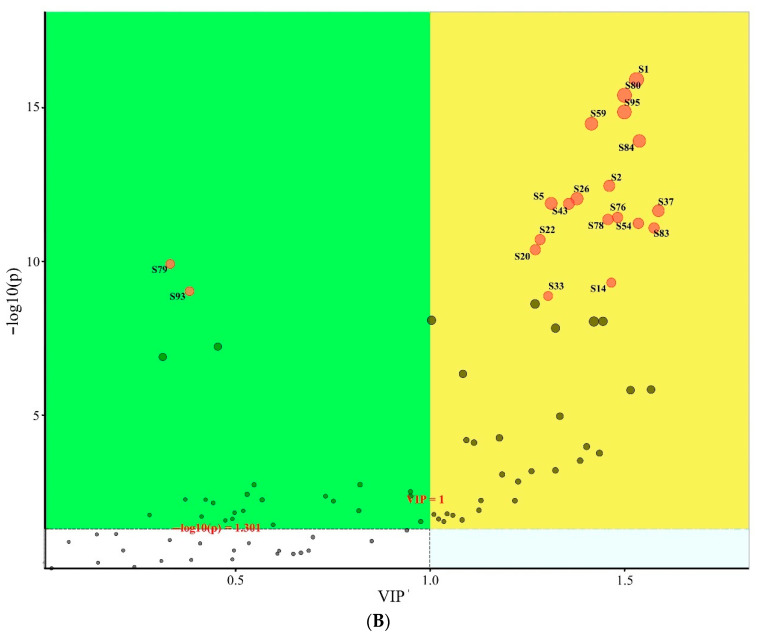
(**A**) PCA results of volatile compounds in MPR samples with different processing. C: Control group; T1: test group 1 (using the improved technology of Sichuan spicy flavor formula); T2: test group 2 (using the improved technology of halogen flavor formula). The data are from 6 repeats of each experimental group. (**B**) PLS-DA diagram of 115 volatile compounds. C: Control group; T1: test group 1 (using the improved technology of Sichuan spicy flavor formula); T2: test group 2 (using the improved technology of halogen flavor formula). The red circles (VIP > 1 and *p* < 0.05) are the volatile compounds that have changed significantly during the fermentation and different process of making MPR. The higher the VIP value is, the greater the difference. S1: Cyclopropane, ethylidene-, S2: Cyclopropane, S5: 2,7-dimethyloctane, S14: Tridecane, S20: 1-Pentanol, S22: Trans-3-Hexen-1-ol, S26: 5-Hepten-2-ol, 6-methyl-, S33: 2,3-Butanediol, S37: 3-Hexenal, S43: 3-Methoxycinnamaldehyde, S54: Methyl-, nonyl-, ketone, S59: 2-butynoic acid, methyl ester, S76: Acetic acid, isoamyl ester, S78: Pent-4-en-1-yl propyl carbonate, S80: 4-Methyl-4-vinylbutyrolactone, S83: Acetic acid, linalyl, S84: Acetic acid, methoxy-, S95: Acetic acid.

PCA and PLS-DA were established to explore the differences in the 114 detected volatile components of the samples with different MPR and to gain an in-depth understanding of the dynamic changes in these substances during the fermentation and other different processes. VIP ≥ 1 is the screening standard for common differential metabolites. The PCA of the volatile components in the samples is shown in Figure 3A. Principal components 1 and 2 distinguished samples from each group. Each group had good repeatability, and significant differences were observed between groups. The red column in Figure 3B suggests the differences in substances among the three experience groups. A total of 18 different volatile substances (VIP ≥ 1) were obtained, including alkanes (4): Cyclopropane, ethylidene-, Cyclopropane, 2,7-dimethyloctane, Tridecane; esters (5): 2-butynoic acid, methyl ester, Acetic acid, isoamyl ester, Pent-4-en-1-yl propyl carbonate, 4-Methyl-4-vinylbutyrolactone, Acetic acid, linalyl; alcohols (4): 1-Pentanol, Trans-3-Hexen-1-ol, 5-Hepten-2-ol, 6-methyl-, 2,3-Butanediol; ketones (1): Methyl-, nonyl-, ketone; acids (2): Acetic acid, methoxy-, Acetic acid; aldehydes (2): 3-Hexenal, 3-Methoxycinnamaldehyde.

### 3.8. Correlation Analysis between Processing and Volatiles

Heatmaps can visually show the difference in the content of volatile substances and specific components. The difference in the mean size of each component in the figure is indicated by a different color shade. Darker colors mean more content, while lighter colors suggest less content; the darkest color is a unique ingredient between different varieties.

**Figure 4:**
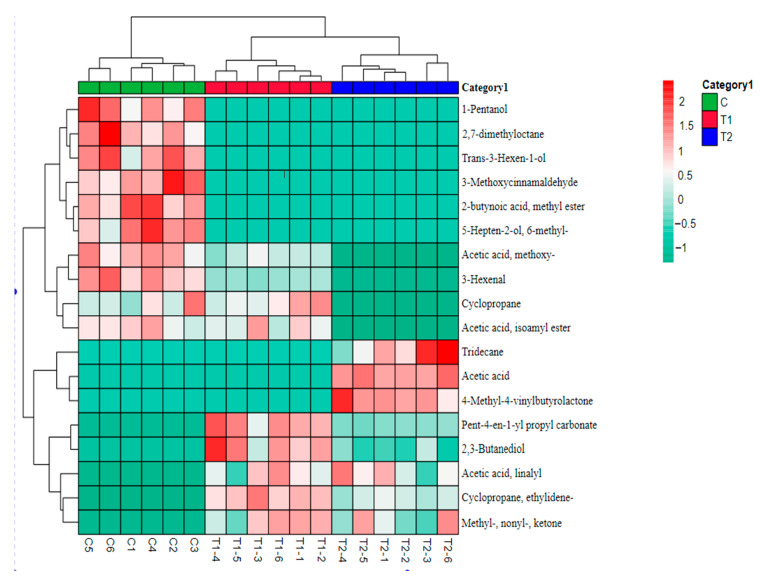
Spearman correlation analysis heatmap between different processing and the volatile components that have changed significantly in MPR. C: Control group; T1: test group 1 (using the improved technology of Sichuan spicy flavor formula); T2: test group 2 (using the improved technology of halogen flavor formula).

As shown in Figure 4, the composition and content of different metabolites in the three groups of MPR are significantly different. It shows that these 18 metabolites had an important contribution to the flavor between different groups. Among them, only aldehydes had a low threshold, which contributed greatly to the overall flavor. After the improvement in formula and processing technology, the specific components of volatile substances in T1 and T2 changed significantly compared with C. 

### 3.9. The Influence of Process Optimization on the Sensory Evaluation of MPR

**Figure 5:**
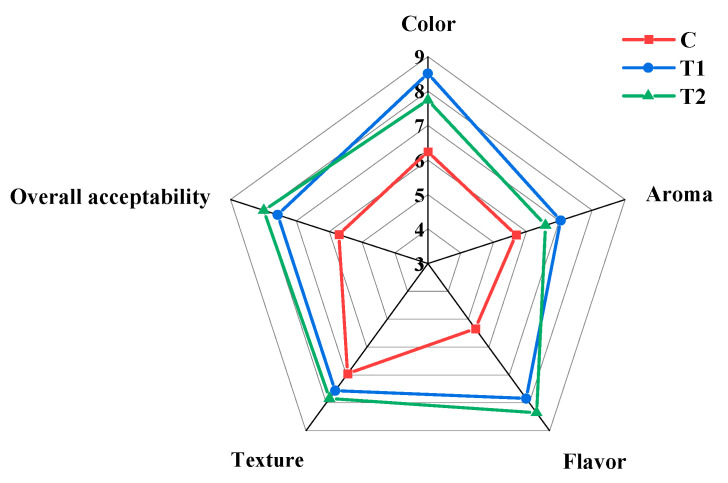
Sensory evaluation of MPR samples with different processing. C: Control group; T1: test group 1 (using the improved technology of Sichuan spicy flavor formula); T2: test group 2 (using the improved technology of halogen flavor formula).

The scores of sensory attributes (color, aroma, flavor, texture and overall acceptability) of T1 and T2 were significantly higher than C (*p* < 0.05) (Figure 5). The higher color score of T1 and T2 may result from kneading techniques and the addition of pepper. It has been noticed that the aroma, flavor and overall acceptability scores of T1 and T2 were significantly (*p* < 0.05) increased compared to C, which may be explained on the basis of improvements in process and formulation to reduce pH and hardness and to increase the water content. Meat aroma and flavor are directly related to the flavor substances, such as volatile compounds, FAAs and free fatty acids profile, present in the meat [59]. Combined with the data on volatile compounds and free amino acids, the increase in meat flavor and aroma of T1 and T2 could be attributed to more FAAs and fewer acids. Meanwhile, the higher tenderness scores of T1 and T2 may be explained by kneading and the addition of exogenous proteases so that the structure of proteins changed, which made it easier to degrade in the marinating process.

## 4. Conclusions

The present study has investigated the effects of different processes and formulations on MPR by controlling the fermentation conditions (fermentation time 10 d, fermentation temperature 14 °C), adjusting the fat-to-lean ratio of meat (lean: fat was 8:2) and adding papain (addition amount 0.035%), enzymatic tenderization and tumbling technology. By improving different processes of making MPR, the color, acidity, yield of finished products and tenderness of MPR have been improved, which led to changes in the flavor profile and quality of MPR. Furthermore, the scores of sensory attributes and the content of TFAAs in T1 and T2 underwent significant changes; specifically, the content of umami amino acids increased significantly. The characterization and identification of flavor substances by SPME combined with GC–MS showed distinct differences in the composition of the three groups. Therefore, the processes and formulations had a significant impact on the overall quality improvement of MPR. These findings have increased our understanding of the flavor characteristics of MPR under different conditions and formulations during its processing. The study may provide a reference and rationale for the improvement and evaluation of fermented meat. However, during the fermentation process, the change in bacterial and fungal community structures and the involvement of bacteria or fungi play a vital role in the flavor formation mechanisms of fermented meat products. Hence, further work should focus on the dominant bacterial community, bacterial flora changes and the relationship of the flavor in the course of fermentation, which is the key to developing an industry of traditional fermented meat.

## Figures and Tables

**Table 1:** Sample preparation process formula table of MPR.

Formula	Groups		
	C	T1	T2
Salt	4.00%	2.00%	2.00%
Rice wine	0.75%	0.75%	0.75%
Red Yeast Rice	0.30%	0.44%	0.44%
Isolated Soy Protein	N.	1.50%	1.50%
Potato modified starch	N.	3.00%	3.00%
Sodium Alginate	N.	0.18%	0.18%
Hexametaphosphate	N.	0.21%	0.21%
Sodium Nitrite	0.01%	N.	N.
Spice addition amount	0.40%	0.15%	0.15%
Chili	N.	0.60%	N.
Ginger	N.	0.50%	0.50%
Star anise	0.60%	0.10%	0.35%
Cinnamon	0.20%	N.	0.015%
Pepper	0.4%	0.3%	0.22%
Tsaoko	0.60%	0.10%	0.04%
Clove	0.20%	N.	0.04%
Angelica dahurica	N.	0.10%	0.22%
Bay leaf	N.	N.	0.01%
Cumin	N.	N.	0.02%
Amomum	N.	0.10%	0.15%
Nutmeg	N.	0.10%	0.18%

C: Control group; T1: test group 1 (using the improved technology of Sichuan spicy flavor formula); T2: test group 2 (using the improved technology of halogen flavor formula); N.: Not added.

**Table 2:** The effect of the improvement in pH, water activity, water content and color of MPR.

Physical and Chemical Indicators	C	T1	T2	Sig.
pH	4.26 ± 0.01 ^c^	5.54 ± 0.03 ^b^	5.79 ± 0.02 ^a^	**
Aw	0.96 ± 0.01 ^a^	0.95 ± 0.01 ^a^	0.96 ± 0.01 ^a^	ns
Moisture content (%)	56.05 ± 0.49 ^c^	60.32 ± 1.13 ^a^	59.18 ± 0.83 ^b^	**
L*	58.70 ± 1.68 ^a^	47.42 ± 0.47 ^c^	53.28 ± 0.41 ^b^	**
a*	14.49 ± 0.25 ^c^	24.11 ± 0.86 ^a^	21.57 ± 0.45 ^b^	**
b*	13.99 ± 0.13 ^c^	20.85 ± 1.28 ^a^	15.80 ± 0.30 ^b^	**
Cooking loss rate (100%)	18.21% ± 1.76 ^a^	8.79% ± 0.40 ^c^	9.80% ± 0.73 ^b^	**
Yield (100%)	75.23% ± 1.37 ^b^	85.91% ± 0.25 ^a^	86.03% ± 0.47 ^a^	**

C: Control group; T1: test group 1 (using the improved technology of Sichuan spicy flavor formula); T2: test group 2 (using the improved technology of halogen flavor formula). The results are expressed as mean ± standard deviation (*n* = 3). ^a–c^ within the same row, different lowercase letters indicate significant differences (*p* < 0.05). Sig. is the significance regarding the MPR treatment. ns: not significant (*p* > 0.05); **: very important (*p* < 0.01).

**Table 3:** The improvement in the texture of MPR.

Texture Index	C	T1	T2	Sig.
Hardness (g)	31,729.96 ± 1798.13 ^a^	9053.36 ± 522.11 ^b^	9096.49 ± 486.25 ^b^	**
Elasticity	0.93 ± 0.04 ^a^	0.62 ± 0.02 ^b^	0.67 ± 0.03 ^b^	*
Cohesion	0.49 ± 0.00 ^a^	0.29 ± 0.01 ^c^	0.38 ± 0.01 ^b^	**
Chewiness	6906.15 ± 463.92 ^a^	2341.48 ± 153.38 ^b^	2653.33 ± 229.63 ^b^	**
Resilience	0.17 ± 0.01 ^a^	0.16 ± 0.01 ^a^	0.17 ± 0.02 ^a^	ns
Shear force (N)	8771.12 ± 643.97 ^a^	4826.65 ± 592.92 ^b^	4962.39 ± 663.91 ^b^	**

C: Control group; T1: test group 1 (using the improved technology of Sichuan spicy flavor formula); T2: test group 2 (using the improved technology of halogen flavor formula). The results are expressed as mean ± standard deviation (*n* = 3). ^a–c^ within the same row, different lowercase letters indicate significant differences (*p* < 0.05). Sig. is the significance regarding the MPR treatment. ns: not significant (*p* > 0.05); *: important (*p* < 0.05); **: very important (*p* < 0.01).

**Table 4:** The effect of the content of FAAs (mg/g) in MPR.

Taste Characteristics	FAAs	C	T1	T2	Sig.
Umami Amino Acids	Glutamic acid (Glu)	0.74 ± 0.03 ^c^	1.49 ± 0.06 ^b^	1.66 ± 0.19 ^a^	**
	Aspartic acid (Asp)	0.09 ± 0.01 ^c^	0.13 ± 0.01 ^b^	0.15 ± 0.01 ^a^	**
Sweet amino acid	Alanine (Ala)	0.01 ± 0.01 ^c^	0.03 ± 0.01 ^a^	0.02 ± 0.01 ^b^	**
	Glycine (Gly)	0.07 ± 0.01 ^a^	0.04 ± 0.01 ^c^	0.06 ± 0.01 ^b^	**
	Serine (Ser)	0.12 ± 0.01 ^a^	0.03 ± 0.01 ^c^	0.08 ± 0.01 ^b^	**
	Threonine (Thr)	0.12 ± 0.02 ^a^	0.03 ± 0.01 ^c^	0.09 ± 0.01 ^b^	**
Bitter amino acid	Arginine (Arg)	0.14 ± 0.02 ^a^	0.05 ± 0.01 ^c^	0.08 ± 0.02 ^b^	**
	Isoleucine (Ile)	0.08 ± 0.01 ^a^	0.03 ± 0.01 ^c^	0.04 ± 0.01 ^b^	**
	Leucine (Leu)	0.07 ± 0.01 ^a^	0.03 ± 0.01 ^b^	0.04 ± 0.01 ^b^	**
	Methionine (Met)	0.17 ± 0.01 ^a^	0.06 ± 0.01 ^b^	0.06 ± 0.01 ^b^	**
	Phenylalanine (Phe)	0.13 ± 0.01 ^a^	0.05 ± 0.03 ^b^	0.11 ± 0.01 ^a^	**
	Tyrosine (Tyr)	0.01 ± 0.03 ^a^	0.02 ± 0.01 ^a^	0.02 ± 0.01 ^a^	ns
	Valine (Val)	0.12 ± 0.02 ^a^	0.08 ± 0.02 ^b^	0.08 ± 0.02 ^b^	**
Odorless amino acids	Lysine (Lys)	0.02 ± 0.01 ^c^	0.08 ± 0.01 ^b^	0.16 ± 0.03 ^a^	**
	Proline (Pro)	0.06 ± 0.02 ^a^	0.06 ± 0.01 ^a^	0.07 ± 0.01 ^a^	ns
Total content	Total FAAs	1.96 ± 0.05 ^c^	2.23 ± 0.08 ^b^	2.74 ± 0.19 ^a^	**
	Umami Amino Acids	0.83 ± 0.02 ^c^	1.63 ± 0.06 ^b^	1.81 ± 0.09 ^a^	**
	Sweet amino acid	0.32 ± 0.02 ^a^	0.13 ± 0.01 ^c^	0.25 ± 0.01 ^b^	**
	Bitter amino acid	0.72 ± 0.03 ^a^	0.33 ± 0.03 ^c^	0.44 ± 0.13 ^b^	**
	Odorless amino acids	0.09 ± 0.01 ^c^	0.14 ± 0.01 ^b^	0.24 ± 0.03 ^a^	**

C: Control group; T1: test group 1 (using the improved technology of Sichuan spicy flavor formula); T2: test group 2 (using the improved technology of halogen flavor formula). The results are expressed as mean ± standard deviation (*n* = 3). ^a–c^ within the same row, different lowercase letters indicate significant differences (*p* < 0.05). Sig. is the significance regarding the MPR treatment. ns: not significant (*p* > 0.05); **: very important (*p* < 0.01).

**Table 5:** The FAAs TAV of MPR with different process conditions.

Taste Characteristics	FAAs	Threshold (mg/g)	TAV			Sig.
			C	T1	T2	
Umami Amino Acids	Glu	0.3	2.47 ± 0.03 ^c^	4.97 ± 0.06 ^b^	5.53 ± 0.19 ^a^	**
	Asp	1	0.09 ± 0.01 ^c^	0.13 ± 0.01 ^b^	0.15 ± 0.01 ^a^	**
	Total	—	2.56 ± 0.02 ^c^	5.10 ± 0.06 ^b^	5.68 ± 0.09 ^a^	**
Sweet amino acid	Ala	0.6	0.02 ± 0.01 ^c^	0.05 ± 0.01 ^a^	0.03 ± 0.03 ^b^	**
	Gly	1.3	0.05 ± 0.02 ^a^	0.03 ± 0.01 ^b^	0.05 ± 0.01 ^a^	**
	Ser	1.5	0.08 ± 0.01 ^a^	0.02 ± 0.03 ^c^	0.05 ± 0.01 ^b^	**
	Thr	2.6	0.05 ± 0.01 ^a^	0.01 ± 0.02 ^c^	0.03 ± 0.01 ^b^	**
	Total	—	0.2 ± 0.01 ^a^	0.11 ± 0.02 ^c^	0.17 ± 0.02 ^b^	**
Bitter amino acid	Arg	0.5	0.28 ± 0.01 ^a^	0.10 ± 0.03 ^c^	0.16 ± 0.01 ^b^	**
	Ile	0.9	0.09 ± 0.01 ^a^	0.03 ± 0.02 ^b^	0.04 ± 0.02 ^b^	**
	Leu	1.9	0.04 ± 0.02 ^a^	0.02 ± 0.01 ^b^	0.02 ± 0.01 ^b^	**
	Met	0.3	0.37 ± 0.01 ^a^	0.20 ± 0.01 ^b^	0.20 ± 0.01 ^b^	**
	Phe	0.9	0.14 ± 0.02 ^a^	0.06 ± 0.01 ^b^	0.12 ± 0.01 ^a^	**
	Val	0.4	0.30 ± 0.02 ^a^	0.20 ± 0.01 ^b^	0.20 ± 0.01 ^b^	**
	Total	—	1.22 ± 0.02 ^a^	0.61 ± 0.01 ^c^	0.74 ± 0.02 ^b^	**
Odorless amino acids	Lys	0.5	0.04 ± 0.03 ^c^	0.16 ± 0.02 ^b^	0.32 ± 0.01 ^a^	**
	Pro	3	0.02 ± 0.01 ^a^	0.02 ± 0.02 ^a^	0.02 ± 0.01 ^a^	ns
	Total	—	0.06 ± 0.02 ^c^	0.18 ± 0.02 ^b^	0.34 ± 0.01 ^a^	**

C: Control group; T1: test group 1 (using the improved technology of Sichuan spicy flavor formula); T2: test group 2 (using the improved technology of halogen flavor formula). The results are expressed as mean ± standard deviation (*n* = 3). “—”: No. ^a–c^ within the same row, different lowercase letters indicate significant differences (*p* < 0.05). Sig. is the significance regarding the MPR treatment. ns: not significant (*p* > 0.05); **: very important (*p* < 0.01).

**Table 6:** The effect of the improvement on the relative content of volatile compounds (%) in MPR.

NO.	Volatile Compounds	C	T1	T2	Sig.
	Alkanes	5.83 ± 0.18 ^b^	10.14 ± 1.55 ^a^	7.00 ± 0.54 ^b^	**
1	Cyclopropane, ethylidene-	ND	4.18 ± 1.03 ^a^	2.74 ± 0.32 ^b^	**
2	Cyclopropane	2.20 ± 0.21 ^a^	2.41 ± 0.40 ^a^	ND	**
3	Dodecane, 4-methyl-	0.18 ± 0.23 ^a^	0.02 ± 0.01 ^a^	ND	ns
4	Dodecane	0.80 ± 0.07 ^a^	0.29 ± 0.03 ^b^	0.27 ± 0.02 ^b^	**
5	2,7-dimethyloctane	0.24 ± 0.03	ND	ND	**
6	Tridecane, 5-methyl-	0.02 ± 0.01 ^ab^	0.01 ± 0.01 ^b^	0.12 ± 0.09 ^a^	ns
7	Tridecane, 3-methyl-	0.09 ± 0.01 ^a^	0.04 ± 0.01 ^b^	0.04 ± 0.01 ^b^	**
8	Tetradecane	0.10 ± 0.01 ^a^	0.07 ± 0.01 ^b^	0.05 ± 0.01 ^b^	**
9	n-Hexadecane	0.04 ± 0.01 ^a^	0.07 ± 0.04 ^a^	0.05 ± 0.01 ^a^	ns
10	n-Pentadecane	0.03 ± 0.01 ^b^	0.06 ± 0.01 ^a^	0.05 ± 0.02 ^ab^	ns
11	Pentadecane, 3-methyl-	0.01 ± 0.01	ND	ND	ns
12	Nonane, 3-methyl-	0.13 ± 0.04 ^a^	0.05 ± 0.01 ^b^	0.13 ± 0.03 ^a^	*
13	Decane	1.97 ± 0.04 ^a^	1.00 ± 0.14 ^c^	1.19 ± 0.09 ^b^	**
14	2,2,7,7-Tetramethyloctane	ND	1.95 ± 0.22 ^a^	2.11 ± 0.11 ^a^	**
15	Tridecane	ND	ND	0.20 ± 0.01	**
16	Bicyclo[3.1.1]heptane, 6,6-dimethyl-2-methylene-, (1S)-	0.03 ± 0.01 ^b^	ND	0.06 ± 0.01 ^a^	**
	Alcohols	34.43 ± 2.02 ^a^	36.80 ± 2.23 ^a^	35.30 ± 2.26 ^a^	ns
17	Ethanol	7.11 ± 0.63 ^a^	5.07 ± 0.51 ^b^	4.56 ± 0.60 ^b^	**
18	Propanol	0.20 ± 0.05	ND	ND	**
19	1,5-hexadiene-3-ol	0.06 ± 0.01	ND	ND	**
20	(+)-3-Methyl-2-butanol	0.22 ± 0.16 ^a^	0.22 ± 0.02 ^a^	ND	*
21	Pentanol	4.78 ± 0.37 ^a^	5.05 ± 0.59 ^a^	4.23 ± 0.63 ^a^	ns
22	1-Pentanol	0.14 ± 0.01	ND	ND	**
23	1-Pentanol, 4-methyl-	ND	0.01 ± 0.01	ND	ns
24	Trans-3-Hexen-1-ol	0.16 ± 0.01	ND	ND	**
25	4-Hexen-1-ol, (E)-	8.93 ± 0.67 ^b^	13.01 ± 3.27 ^a^	12.90 ± 1.48 ^a^	ns
26	2-Heptanol	0.34 ± 0.05 ^a^	0.17 ± 0.02 ^b^	0.21 ± 0.01 ^b^	**
27	1-Penten-3-ol	0.06 ± 0.01 ^b^	0.15 ± 0.13 ^ab^	0.34 ± 0.16 ^a^	ns
28	(R)-(-)-3-Methyl-2-butanol	ND	0.24 ± 0.02	ND	**
29	5-Hepten-2-ol, 6-methyl-	2.10 ± 0.14	ND	ND	**
30	2-phenyl-2-butanol	0.02 ± 0.01	ND	ND	**
31	Linalool	7.48 ± 0.88 ^b^	9.49 ± 0.42 ^a^	9.91 ± 0.63 ^a^	**
32	Terpinen-4-ol	0.54 ± 0.07 ^b^	0.35 ± 0.04 ^c^	0.91 ± 0.02 ^a^	**
33	Bicyclo[2.2.1]heptan-2-ol, 2,3,3-trimethyl-	0.02 ± 0.01 ^b^	0.03 ± 0.01 ^ab^	0.05 ± 0.01 ^a^	*
34	α-Methyl-α-[4-methyl-3-pentenyl]oxiranemethanol	0.95 ± 0.14 ^a^	0.48 ± 0.09 ^b^	0.47 ± 0.03 ^b^	**
35	Nerol	0.44 ± 0.08 ^a^	0.27 ± 0.02 ^b^	0.03 ± 0.01 ^c^	**
36	Nerolidol	0.02 ± 0.01 ^a^	0.01 ± 0.01 ^a^	0.02 ± 0.01 ^a^	ns
37	2,3-Butanediol	ND	0.30 ± 0.07 ^a^	0.07 ± 0.01 ^b^	**
38	2,3-Butanediol, [S-(R*,R*)]-	ND	0.57 ± 0.04	ND	**
39	2-Cyclohexen-1-ol, 3-methyl-6-(1-methylethyl)-, trans-	0.17 ± 0.03 ^b^	0.32 ± 0.05 ^a^	ND	**
40	2-Cyclohexen-1-ol, 1-methyl-4-(1-methylethyl)-, trans-	0.30 ± 0.01 ^b^	0.45 ± 0.09 ^b^	1.21 ± 0.14 ^a^	**
41	2-Isopropyl-1,3-propanediol	ND	0.06 ± 0.01	ND	**
42	5-Isopropyl-2-methylbicyclo[3.1.0]hexan-2-ol	0.26 ± 0.02 ^b^	0.34 ± 0.01 ^a^	0.31 ± 0.07 ^a^	ns
43	Phenylethyl Alcohol	0.14 ± 0.01 ^ab^	0.20 ± 0.01 ^a^	0.10 ± 0.07 ^b^	ns
	Aldehydes	2.56 ± 0.21 ^a^	2.86 ± 0.46 ^a^	2.65 ± 0.62 ^a^	ns
44	n-Hexanal	0.34 ± 0.01 ^a^	0.44 ± 0.16 ^a^	0.46 ± 0.14 ^a^	ns
45	3-Hexenal	0.62 ± 0.01 ^a^	0.29 ± 0.04 ^b^	ND	**
46	2,4-Hexadienal, (E,E)-	0.44 ± 0.22 ^b^	1.55 ± 0.55 ^a^	1.49 ± 0.60 ^ab^	ns
47	2-Hexadienal, (E)-	0.05 ± 0.01	ND	ND	**
48	Benzaldehyde	0.07 ± 0.04 ^a^	0.04 ± 0.01 ^a^	0.03 ± 0.01 ^a^	ns
49	Benzeneacetaldehyde	ND	0.01 ± 0.01 ^a^	0.01 ± 0.01 ^a^	ns
50	2,6-Octadienal, 3,7-dimethyl-, (E)-	0.05 ± 0.01 ^a^	0.02 ± 0.01 ^a^	0.03 ± 0.01 ^a^	*
51	Cinnamaldehyde	0.04 ± 0.01 ^a^	0.06 ± 0.01 ^a^	0.08 ± 0.01 ^a^	**
52	3-Methoxycinnamaldehyde	0.86 ± 0.04	ND	ND	**
53	Hexadedehyde	0.02 ± 0.01	ND	ND	**
54	Benzaldehyde, 4-(1-methylethyl)-	0.02 ± 0.01 ^b^	0.22 ± 0.01 ^a^	0.19 ± 0.02 ^a^	**
55	3-Hexenal, (Z)-	ND	0.19 ± 0.01 ^a^	0.16 ± 0.05 ^a^	**
56	2-heptanal	0.05 ± 0.01 ^a^	0.05 ± 0.01 ^a^	0.06 ± 0.01 ^a^	ns
57	Benzaldehyde, 4-methoxy-	ND	ND	0.14 ± 0.01	**
	Ketones	1.69 ± 0.21 ^b^	2.95 ± 0.37 ^a^	1.71 ± 0.03 ^b^	**
58	2,5-Hexanedione	0.02 ± 0.01	ND	ND	*
59	Bicyclo[3.1.1]heptan-2-one, 6,6-dimethyl-, (1 R)-	0.69 ± 0.08 ^a^	0.72 ± 0.02 ^a^	ND	**
60	Thujone	0.28 ± 0.02 ^b^	0.51 ± 0.03 ^a^	0.43 ± 0.03 ^a^	**
61	Bicyclo[3.1.0]hexan-3-one, 4-methyl-1-(1-methylethyl)-, [1S-(1α,4β,5α)]-	0.20 ± 0.01 ^a^	0.22 ± 0.01 ^a^	0.13 ± 0.01 ^b^	**
62	Piperitone	0.42 ± 0.09 ^a^	0.24 ± 0.04 ^b^	0.14 ± 0.01 ^b^	**
63	1-Propanone, 1-(4-methoxyphenyl)-	0.04 ± 0.01	ND	ND	**
64	4’,6’-Dimethoxy-2’,3’-dimethylacetophenone	0.04 ± 0.01 ^a^	0.02 ± 0.01 ^ab^	0.01 ± 0.01 ^b^	*
65	Methyl-, nonyl-, ketone	ND	1.24 ± 0.37 ^a^	1.01 ± 0.03 ^b^	**
	Esters	25.27 ± 0.50 ^a^	14.56 ± 1.69 ^b^	18.92 ± 0.79 ^b^	*
66	Acrylic acid, Isoamyl ester	0.16 ± 0.12 ^b^	0.48 ± 0.14 ^a^	0.25 ± 0.12 ^ab^	ns
67	Acetic acid, propyl ester	0.15 ± 0.03 ^a^	0.09 ± 0.03 ^b^	0.01 ± 0.01 ^c^	**
68	Butyric acid, methyl ester	0.01 ± 0.01 ^a^	0.01 ± 0.01 ^a^	ND	ns
69	Butyric acid, ethyl ester	0.32 ± 0.02 ^a^	0.24 ± 0.03 ^b^	0.31 ± 0.01 ^a^	**
70	2-butynoic acid, methyl ester	14.94 ± 1.89	ND	ND	**
71	Ethyl acetate	6.96 ± 1.24 ^b^	7.37 ± 1.52 ^a^	7.41 ± 0.68 ^a^	ns
72	2,4-Pentadienoic acid, 1-cyclopenten-3-on-1-yl ester	ND	ND	1.69 ± 0.28	**
73	Propanedioic acid, oxo-, bis(1-methylethyl) ester	ND	3.08 ± 0.81	ND	**
74	Diisopropyl 2-oxomalonate	0.04 ± 0.01	ND	ND	**
75	Hexanoic acid, methyl ester	0.04 ± 0.02 ^a^	0.08 ± 0.01 ^a^	0.12 ± 0.01 ^a^	**
76	Isocaproic acid, ethyl ester	0.02 ± 0.01	ND	ND	**
77	n-Hexanoic acid, ethyl ester	0.30 ± 0.02 ^a^	0.19 ± 0.01 ^b^	0.17 ± 0.01 ^b^	**
78	Acetic acid, hexyl ester	0.01 ± 0.01 ^a^	0.01 ± 0.01 ^a^	ND	ns
79	Sorbic acid, methyl ester	0.08 ± 0.11 ^a^	ND	0.01 ± 0.01 ^a^	ns
80	Octanoic acid, methyl ester	0.06 ± 0.01 ^a^	0.04 ± 0.02 ^ab^	0.02 ± 0.01 ^b^	*
81	Benzoic acid, ethyl ester	0.07 ± 0.04 ^b^	0.07 ± 0.01 ^b^	0.25 ± 0.02 ^a^	**
82	Octanoic acid, ethyl ester	0.94 ± 0.14 ^a^	0.48 ± 0.09 ^b^	0.47 ± 0.07 ^b^	**
83	Acetic acid, geranyl ester	0.13 ± 0.02 ^a^	0.10 ± 0.02 ^a^	0.13 ± 0.01 ^a^	ns
84	Decanoic acid, ethyl ester	0.32 ± 0.06 ^a^	0.17 ± 0.06 ^b^	0.10 ± 0.01 ^b^	**
85	Tetradecanoic acid, ethyl ester	0.02 ± 0.01 ^a^	0.02 ± 0.01 ^a^	0.01 ± 0.01 ^a^	ns
86	9-Octadecenoic acid, ethyl ester	0.02 ± 0.01 ^a^	0.01 ± 0.01 ^a^	0.01 ± 0.01 ^a^	ns
87	Glycidyl palmitoleate	0.01 ± 0.01 ^b^	0.04 ± 0.01 ^a^	0.02 ± 0.01 ^b^	**
88	Hydroxymethyl 2-hydroxy-2-methylpropionate	ND	0.37 ± 0.08 ^a^	0.25 ± 0.04 ^a^	**
89	Propionic acid, ethyl ester	0.17 ± 0.03 ^a^	0.11 ± 0.01 ^a^	ND	**
90	Acetic acid, isoamyl ester	0.48 ± 0.06 ^a^	0.46 ± 0.04 ^a^	ND	**
91	Butanoic acid, 3-hydroxy-, ethyl ester	ND	0.07 ± 0.01	ND	**
92	Pent-4-en-1-yl propyl carbonate	ND	0.33 ± 0.06 ^a^	0.13 ± 0.02 ^b^	**
93	Acetic acid, 4-terpineol, ester	ND	0.17 ± 0.02	ND	
94	4-Methyl-4-vinylbutyrolactone	ND	ND	2.16 ± 0.04	**
95	Lactic acid, ethyl ester, (L)-	ND	0.20 ± 0.01 ^a^	0.10 ± 0.01 ^b^	**
96	5-Oxotetrahydrofuran-2-carboxylic acid, ethyl ester	ND	ND	2.12 ± 0.02	**
97	3,3-dimethyl-, 1-butyl acid, ester	ND	ND	2.11 ± 0.01	**
98	Acetic acid, linalyl, ester	ND	0.40 ± 0.04 ^a^	0.41 ± 0.03 ^a^	**
	Acids	7.12 ± 0.71 ^a^	4.71 ± 0.19 ^b^	3.48 ± 0.05 ^b^	**
99	Acetic acid, methoxy-	3.97 ± 0.21 ^a^	2.45 ± 0.11 ^b^	ND	**
100	Butanoic acid	0.45 ± 0.12 ^a^	0.45 ± 0.15 ^a^	0.16 ± 0.08 ^b^	*
101	Isovaleric acid	0.04 ± 0.01 ^b^	0.09 ± 0.02 ^a^	0.03 ± 0.01 ^b^	**
102	Sorbic acid	1.43 ± 0.47 ^a^	1.04 ± 0.10 ^a^	0.41 ± 0.02 ^b^	*
103	Octanoic acid	0.58 ± 0.04	ND	ND	**
104	n-Decanoic acid	0.12 ± 0.03 ^ab^	0.10 ± 0.01 ^b^	0.15 ± 0.01 ^a^	ns
105	Myristic acid	0.01 ± 0.01	ND	ND	ns
106	Palmitic acid	0.01 ± 0.01 ^a^	0.01 ± 0.01 ^a^	0.01 ± 0.01 ^a^	ns
107	Oleic Acid	0.34 ± 0.46 ^a^	0.34 ± 0.02 ^a^	0.17 ± 0.03 ^a^	ns
108	Acetic acid, (acetyloxy)-	ND	0.10 ± 0.04	ND	**
109	L-Lactic acid	0.15 ± 0.01 ^a^	0.15 ± 0.01 ^a^	ND	**
110	Acetic acid	ND	ND	2.55 ± 0.12	**
	Other volatile compounds	3.31 ± 0.06 ^a^	3.12 ± 0.25 ^a^	3.26 ± 0.18 ^a^	ns
111	Methyl mercaptan	0.02 ± 0.01	ND	ND	**
112	Eucalyptol	3.21 ± 0.27 ^a^	3.11 ± 0.76 ^a^	2.03 ± 0.25 ^b^	*
113	2-Furanmethanol, 5-ethenyltetrahydro-α,α,5-trimethyl-, cis-	ND	ND	1.16 ± 0.05	**
114	Trans-α-bergamotene	0.08 ± 0.05 ^a^	0.01 ± 0.01 ^b^	0.07 ± 0.01 ^ab^	ns

C: Control group; T1: test group 1 (using the improved technology of Sichuan spicy flavor formula); T2: test group 2 (using the improved technology of halogen flavor formula). The results are expressed as mean ± standard deviation (*n* = 3). ^a–c^ within the same row, different lowercase letters indicate significant differences (*p* < 0.05). Sig. is the significance regarding the MPR treatment. ns: not significant (*p* > 0.05); *: important (*p* < 0.05); **: very important (*p* < 0.01).

## Data Availability

All data are contained within the article.
